# Cytokines, GM-CSF and IFNγ administered by priming and post-chemotherapy cycling in recurrent ovarian cancer patients receiving carboplatin

**DOI:** 10.1186/1479-5876-4-16

**Published:** 2006-04-07

**Authors:** Sachin M Apte, Saroj Vadhan-Raj, Lorenzo Cohen, Roland L Bassett, Ilyssa O Gordon, Charles F Levenback, Pedro T Ramirez, Stacie T Gallardo, Rebecca S Patenia, Michael E Garcia, Revathy B Iyer, Ralph S Freedman

**Affiliations:** 1 Department of Gynecologic Oncology, The University of Texas M.D. Anderson Cancer Center, Houston, Texas, USA; 2 Department of Bioimmunotherapy, The University of Texas M.D. Anderson Cancer Center, Houston, Texas, USA; 3 Department of Behavioral Science, The University of Texas M.D. Anderson Cancer Center, Houston, Texas, USA; 4 Department of Biostatistics, The University of Texas M.D. Anderson Cancer Center, Houston, Texas, USA; 5 Department of Diagnostic Radiology, The University of Texas M. D. Anderson Cancer Center, Houston, Texas, USA

## Abstract

**Background:**

Monocyte/macrophages (MO/MA), a polymorphic population of innate immune cells, have the potential to mediate antitumor effects, and may also contribute to protumor effects. A priming and post-chemotherapy schedule of the myeloid cell mobilizing and immune stimulatory growth factor, granulocyte monocyte stimulating factor (GM-CSF, Leukine^®^) and the MO/MA activating cytokine recombinant interferon gamma 1b (rIFN-γ1b, Actimmune^®^) has been developed. The pre- and post-chemotherapy design is based upon known *in vivo *kinetics and immune modulatory effects of these molecules. Carboplatin (Paraplatin^®^) was selected as the cornerstone of treatment of epithelial ovarian cancer (EOC).

**Methods:**

We studied hematopoietic and immunologic effects of GM-CSF and rIFN-γ1b before and after carboplatin in patients with recurrent EOC. Potentially chemotherapy-sensitive patients with recurrent measurable tumors received subcutaneous GM-CSF (starting at 400 μg/day) for 7 days plus subcutaneous rIFN-γ1b (100 μg) on days 5 and 7, before and after intravenous carboplatin (area under the curve of 5). We performed standard hematologic assessment and monitored monocyte (MO), dendritic cell, major cell subset counts, and antibody-dependent cell-mediated cytotoxicity (ADCC) against a Her2neu^+ ^tumor cell line, as well as selected plasma inflammatory cytokine, chemokine and growth factor levels.

**Results:**

Our analysis comprised only the first 3 months of treatment in the initial 25 patients. Relative to pretreatment baseline values, white blood cell, neutrophil, MO, and eosinophil counts increased (*P *≤ .001 for each); the proportion of platelets increased 9 days after the second (*P *≤ .002) and third (*P *≤ .04) carboplatin treatments; and the number of cells in the activated MO subsets CD14+HLA-DR+, CD14+CD64+, and CD14^+^CXCR3^+ ^increased (*P *≤ .04 for each); plasma levels of the proangiogenic interleukins 1α, 6, and 8 were lower (*P *≤ .03 for each); M-CSF, a product of activated MO/MA, was increased on day 9 (*P *= .007); and GM-CSF was increased in plasma after GM-CSF administration (*P *≤ .04). Quality of life measurements were reduced during the GM-CSF/IFN-γ1b cycle while recovering at pre-chemotherapy baseline for FACT-G scores only.

**Conclusion:**

A novel regimen of GM-CSF plus IFN-γ1b administered to 25 EOC patients receiving carboplatin increased myeloid cells, platelets and total activated MO populations during the initial 3 months; however, ADCC responses were not consistently enhanced during this period.

## Introduction

In 2005 there will be an estimated 22,200 new cases of epithelial ovarian cancer (EOC) and 16,210 deaths from it in the United States, where EOC is the fifth most common cause of cancer-related death in women [1]. The 5-year overall survival rate for patients with distant metastases is 30.9% [2]. Platinum is the cornerstone of chemotherapy for EOC and, together with paclitaxel, is the most frequently used first-line therapy [3,4]. Recurrences after first-line chemotherapy are typically treated with carboplatin, liposomal doxorubicin, a taxane, topotecan, gemcitabine, or etoposide [5], either as single agents or in combination; single agents, including carboplatin, generate a response rate of 15% to 30%, and response rates are higher after a treatment-free interval of ≥ 6 months [6]. A 7% improvement in survival was recently reported for carboplatin plus paclitaxel over "conventional" platinum-based chemotherapy for recurrent EOC [7]. It remains unknown, however, whether sequential use of these agents might produce an equivalent or better survival outcome.

Monocytes and macrophages (MO/MAs) are the most common innate cell population in humans. Their role in cancer patients is complex because they may either enhance or impair immunity. Granulocyte-monocyte colony-stimulating factor (GM-CSF) and recombinant interferon gamma 1b (IFN-γ1b) are commercially available cytokines that can modulate MO/MA activity. GM-CSF can mobilize and mature myeloid cells, including MO/MAs and dendritic cells (DCs) [8,9], whereas IFN-γ1b is an activator of MO/MAs [10,11].

At biologically active doses, GM-CSF increases both neutrophil and MO counts to reach a plateau within 5 to 10 days [12]. When administered after chemotherapy, GM-CSF reduces the duration of neutropenia and enhances recovery. Prior studies have also demonstrated that intravenous "priming" with GM-CSF before chemotherapy with anthracycline-based chemotherapy expands the pool of myeloid progenitor cells and induces these cells to become quiescent, which may enhance myeloprotection with shortening the duration of severe neutropenia [13,14].

GM-CSF may also stimulate the immune system by enhancing antitumor effects by either innate or adaptive antitumor immunity [15,16]. Thus, GM-CSF induces destruction of tumor cells *in vitro *by stimulated peripheral blood MOs [17], enhances DC maturation [18,19], and has become an important component of certain vaccine trials. In addition to other applications [20], GM-CSF has shown promising results as an adjuvant in melanoma [15] and in breast cancer [21]. GM-CSF has been combined with other cytokines (*e.g*,. rIFN-α, interleukin 2 [IL-2], and IFN-γ1b) and provided some treatment effects against metastatic renal tumors and melanoma [22,23]. Optimum dosing and scheduling of these combinations have not been determined. The addition of other cytokines could have either synergistic or antagonistic effects on GM-CSF's leukocytic proliferation and maturational properties. GM-CSF alone can expand and prime MOs destined to migrate to tissues as MAs, but other stimuli may be needed to mature or activate these cells more fully. Such stimuli could be provided by other cytokines, such as IFN-γ.

We designed a clinical trial of two immunomodulatory cytokines, GM-CSF and IFN-γ1b, administered both before and after carboplatin treatment in patients with platinum-sensitive EOC that had recurred. We expected that IFN-γ1b (administered during the latter half of the GM-CSF cycle) would enhance the activation of MO/MAs while GM-CSF would exert its proliferative effects on myeloid progenitor cells, minimizing any possible antiproliferative effects from the IFN-γ1b. Here we report the hematopoietic and myeloid immune cell effects during the first three courses of a chemoimmunotherapy regimen that consisted of GM-CSF and IFN-γ1b priming, carboplatin, and post-carboplatin GM-CSF and IFN-γ1b. Detailed analysis of the response and toxicity of the ongoing phase II trial will be reported after the trial's completion.

## Patients and methods

### Patient population

Patients eligible for inclusion in the study had Müllerian carcinoma (primary epithelial ovarian, primary peritoneal, or fallopian tube) that had initially responded to platinum-based chemotherapy but recurred after a treatment-free interval of ≥ 6 months; these tumors were considered "potentially platinum sensitive." Patients were also required to have radiographically or clinically measurable disease and CA-125 determinations. Adequate hematologic, renal, and hepatic function was required as defined by: absolute neutrophil count ≥ 1,500 cells/μL, platelet count ≥ 100,000 cells/μL, serum creatinine ≤ 1.5 mg/dL, serum bilirubin ≤ 1.5 mg/dL, and aspartate transaminase ≤ 2.5 × normal. A Zubrod performance status score of ≤ 2 was also required. Patients could not have received more than two prior chemotherapy regimens (first-line platinum and platinum re-induction were counted as one regimen). Other exclusion criteria included prior immunotherapy, abdominal radiotherapy, active heart or autoimmune disease, inflammatory bowel disease, brain metastases, serum albumin ≤ 3 g/dL, or known hypersensitivity to platinum agents. All patients provided informed consent for participation in this IRB approved study.

### Cytokines

GM-CSF (Leukine^®^, specific activity 5.6 × 10^6 ^IU/mg; Berlex Labs, Seattle, WA) was provided in vials containing either 250 or 500 μg of the lyophilized powder and 40 mg of mannitol, 10 mg of sucrose, and 1.2 mg of tromethamine. The cytokine was reconstituted with 1.0 mL of either bacteriostatic water or sterile water at room temperature.

IFN-γ1b (Actimmune^®^, specific activity 20 × 10^6 ^IU/mg noncovalent form of dimeric protein; InterMune, Brisbane, CA) was provided in 0.5-mL vials containing 100 μg of IFN-γ1b, 20 mg of mannitol, 0.36 mg of sodium succinate, and 0.05 mg of polysorbate-20. The cytokine was reconstituted in 0.5 mL of sterile water at room temperature.

### Treatment regimen (Figure 1)

**Figure 1:**
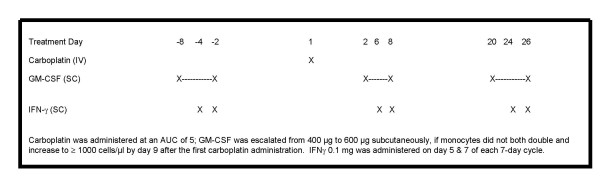
**Treatment Schema**. Schematic of 28-day cycle for the administration of GM-CSF, rIFN-γ1b, and carboplatin.

For each course of treatment, GM-CSF was administered by subcutaneous injection in two 7-day cycles, one preceding and the other one following a fixed intravenous dose of carboplatin (Paraplatin^®^, Bristol-Myers Squibb, New York, NY) at an area under the curve of 5 (Calvert Formula). Carboplatin was administered 48–60 hours after the last dose of GM-CSF. For the post-chemotherapy cycle, GM-CSF was started within 24–36 hours. For the first and second cycles of GM-CSF, the dose started at 400 μg per day and escalated to 600 μg per day if the MO count did not at least double and reach a level of 1,000 cells/μL. The GM-CSF dose could be reduced by 200 μg in response to grade 3 major organ toxicity. IFN-γ1b was administered by subcutaneous injection at a fixed dose of 100 μg on the fifth and seventh days of each 7-day cycle of GM-CSF. No dose escalation of IFN-γ1b was permitted.

For subsequent courses, the pre-chemotherapy GM-CSF/IFN-γ cycle was scheduled to start on day 20. For individual patients who had an absolute neutrophil count of <1,000/μL or a platelet count of <100,000/μL, the subsequent treatment course was delayed until recovery of absolute neutrophil count to ≥ 1,000/μL and platelet count to ≥ 100,000/μL. After recovery, the second dose of IFN-γ1b was omitted on each subsequent cycle of GM-CSF in patients who developed significant neutropenia or thrombocytopenia.

Patients who developed grade 3 allergic reactions were treated with appropriate desensitization. Responses were determined using modified World Health Organization Response Evaluation Criteria in Solid Tumors (RECIST) [24]. Up to three courses of the chemoimmunotherapy regimen were required for the current assessment of hematologic and immunologic effects.

### Hematologic and blood chemistry monitoring

Before the start of each treatment course (*i.e*,. within 10 days before the first GM-CSF dose), the following were checked for each patient: complete blood count, serum creatinine, blood urea nitrogen, total bilirubin, aspartate transaminase, alanine transaminase, electrolytes (Na, K, Cl, and Mg), carbon dioxide, serum albumin, total protein, glucose, alkaline phosphate, lactate dehydrogenase, and CA-125. A complete blood count was also obtained on days -4, 1, 6, 9, 16, 21, 25, and 28 of the first treatment course to monitor the pre and post-chemotherapy GM-CSF/IFN-γ and on days 1, 9, 16, 21, and 28 of the second and third treatment courses.

### Immunologic monitoring

In the 12 patients who consented to optional studies, peripheral blood was examined for the number and activation (or maturation) status of MOs and DCS and for *in vitro *cytotoxicity experiments. Blood specimens were collected on day -8 of the first treatment course before the start of rIFN-γ1b treatment on day -4, before the start of carboplatin treatment on day 1, and 9 days after carboplatin treatment. Additional specimens were collected from patients 3 months after the first treatment course was begun. Peripheral blood (10 ml) was collected in ACD (yellow top) vacutainer tubes The blood was centrifuged, the plasma collected, and the mononuclear cells isolated by Ficoll-Hypaque density gradient separation. In addition, for cytotoxicity studies, MOs were obtained from anonymized normal donors through the blood bank as controls.

#### Flow cytometry

MOs were identified within mononuclear cell preparations by flow cytometry as CD14^bright ^leukocytes or CD14^dim^, CD45^+ ^leukocytes. Mononuclear leukocytes were further characterized for coexpression of HLA-DR, CD64, and in some instances CD32 and CD32B. DCs were identified directly in mononuclear cell preparations of blood as lineage-negative, HLA-DR^+ ^leukocytes by using multidimensional flow cytometry [25,26]. Additionally, three-color labeling was performed to evaluate the proportion and absolute numbers of CD11c^+ ^pre-DC1 cells and CD123^+ ^and pre-DC2 cell subsets [27]. Because the maturation of DCs has been shown to be accompanied by expression of CD83, lineage-negative, HLA-DR^+ ^DCs were also tested for coexpression of this marker. CD16^+ ^and CD16^- ^MOs have been shown to give rise to DCs with different phenotypic and functional properties [28]. Therefore, we also monitored this marker, as well as CD2 (which is also expressed on a subset of MOs with the potential to develop into DCs) [29]. For functional analysis, MOs were isolated from mononuclear cell preparations by negative selection using immunomagnetic cell sorting (Miltenyi Biotech, Bergisch Gladbach, Germany). Staining for the CXCR3+ chemokine receptor was performed on MO of selected patients using a PE-Cy5 labeled mAb in conjunction with a PE labeled CD14 mAb.

### Monocyte-mediated tumor cell cytotoxicity and Antibody-Dependent Cellular Cytotoxicity (ADCC) assays

Enriched MOs from EOC patients undergoing treatment were tested for their ability to induce the cytotoxicity of tumor cells directly or in the presence of an antibody capable of mediating ADCC by using a ^3^H-TdR release assay we developed [30,31]. Briefly, washed SKOV3 human ovarian cancer cells, which are Her2neu+, were labeled with^3^H-labeled thymidine (specific activity of 185 GBq/mmol; 50 μCi per 1.2 × 10^6 ^cells) in 10 mL of RPMI 1640 medium with heat-inactivated 15% fetal bovine serum for 18 to 24 hours at 37°C in a humidified 5% CO_2 _incubator. Target cells were incubated with the humanized IgG1 antibody trastuzumab (Herceptin, Genentech, San Francisco, CA), which recognizes the Her2neu surface antigen, at a concentration of 5 μg/mL for 30 min at 37°C in a shaking water bath. Sensitized target cells were washed once and seeded with MO/MAs at an effects-to-target ratio of 20:1 in 96-well U-bottom microtiter plates. Purified MO were isolated from PBMC by negative selection, using a MO Isolation Kit and a MACS separator (Miltenyi Biotec, Auburn, CA) as per manufacturer's instruction. MOs obtained from non-MCSF stimulated buffy coat samples were utilized as controls as previously described [32] were incubated with murine 2B6 monoclonal antibody (kindly provided by MacroGenics, Rockville, MD) that recognize CD32B at a concentration of 1.5 μg/mL in 1 × 10^6 ^cells for 1 hour. Plates were centrifuged at 1,000 rpm for 1 min and incubated for 60 to 72 hours at 37°C in a humidified 5% CO_2 _incubator.

Plates were spun down at 1,000 rpm for 5 min at 25°C, and cell-free supernatants were transferred to scintillation vials into which 5 mL of scintillation fluid was added (Safety Solve; Research Products International, Mount Prospect, IL). ^3^H-TdR release from target cells was counted in a WinSpectral liquid scintillation counter (EG&G; Wallac, Turku, Finland) for 1 min. The percent ADCC for MO/MAs was calculated as (experimental release - spontaneous release) ÷ (maximum release - spontaneous release) × 100, in which release values are in counts per minutes and experimental release is counts from cell-free culture supernatant from MO/MAs incubated with labeled tumor cells, spontaneous release is counts from cell-free culture supernatant from labeled tumor cells only, and maximum release is counts from cell-free culture supernatant from lysed (5% Triton) labeled tumor cells only.

### Plasma cytokine and chemokine measurements

Patients who consented to optional studies provided samples for measurement of plasma levels of cytokines IL-1α, IL-1β, IL-6, IL-10, and TNF-α and the chemokine CXCL8 (IL-8). Plasma samples were taken on days -8, -4, 1, and 9 of the first course and at 3 months after the first treatment course was begun. We used the Multiplex Bead chemoluminescence assay (Luminex, Austin, TX). The Multiplex Bead Immunoassay is a multiplex assay that uses the Luminex 100 analyzer (Austin, TX) and associated software. Levels of multiple cytokines are measured in small volumes by using fluorescently encoded microspheres labeled with a distinguishable fluorophore that allows the microspheres to be gated to a particular region by the scanner. Antibodies specific for the cytokine or chemokine of interest (Biosource International, Camarillo, CA) are covalently linked to individual beads of a different fluorescent marker, thereby causing the cytokines or chemokine to bind to the specific complementary antibody-coated bead and produce a unique fluorescent signature. The minimum detectable levels using this method are: IL1α, 5 pg/mL; IL-1β, 15 pg/mL; IL-6, 3 pg/mL; IL-10, 5 pg/mL; TNF-α, 10 pg/mL; CXCL8, 3 pg/mL; GM-CSF, 3 pg/mL; and M-CSF, 9 pg/mL.

### Quality of life measurements

We used the Functional Assessment of Chronic Illness Therapy-Biologic Response Modifier (FACT-BRM version 4) to assess changes in patients' quality of life. This questionnaire is a 44-item, cancer-specific measure of health-related quality of life[33] It consists of 27 core items that assess physical, social and family, emotional, and functional well-being (FACT-G)[34] and 17 items that assess disease and treatment-related issues specific to patients receiving cytokine therapy (BRM)[35] Higher scores represent better quality of life. Recent research suggests a change of 7 or more on the FACT-G total score indicates a clinically significant change in quality of life[36] Patients completed the 44-item FACT-BRM on days -8, -4, -1, and 9 of the first treatment course and on days -1 and 9 of the second and third treatment courses.

### Statistics

Wilcoxon rank sum tests were used to evaluate differences in variables between individual time points. No correction was made for multiple testing. Statistical significance was declared for p-values < 5%. SAS 9.1 was used for statistical analyses.

## Results

### Patient characteristics

Characteristics of the first 25 enrolled patients and their tumors and prior treatment regimens are summarized in Table 1. The median age at enrollment into the study was 62 years (range, 49–77 years). Most of the patients had serous EOC that was ovarian in origin.

**Table 1:** Patient and Tumor Characteristics and Prior Treatment (N = 25)

Characteristic or Treatment	No. of Patients
Zubrod performance status score	
0	22
1	1
2	2
Race	
White	22
Hispanic	2
Black	1
Primary tumor site	
Ovary	22
Peritoneum	2
Fallopian tube	1
Histologic subtype	
Serous	22
Clear cell	1
Endometrioid	1
Adenocarcinoma	1
First-line chemotherapy	
Paclitaxel and carboplatin	23
Paclitaxel and cisplatin	1
Cisplatin and doxorubicin	1
Prior reinduction chemotherapy	
Carboplatin	4
High dose with bone marrow transplantation	1
None	20
Prior hormonal treatment	
Yes	10
No	15
Pelvic irradiation	
Yes	1
No	24

### Hematopoietic effects

**Figure 2:**
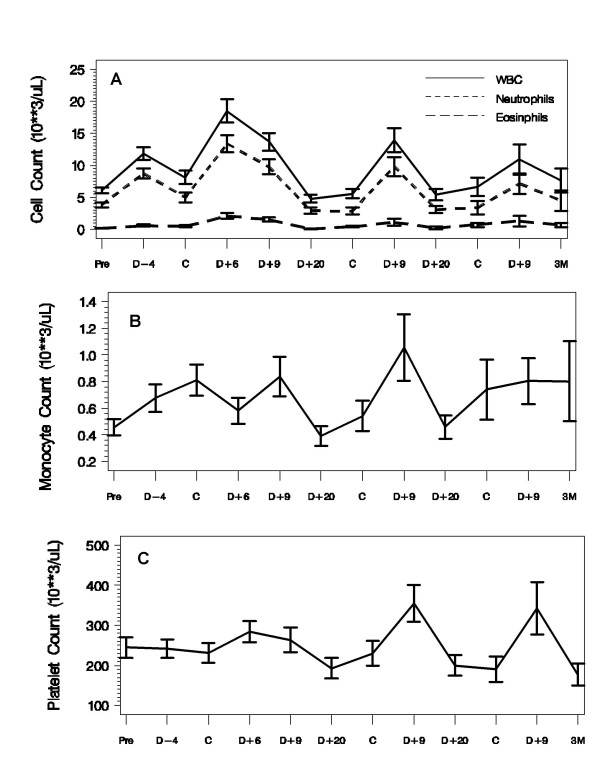
**Hematologic Cell Counts as Function of Time in 25 patients**. "Pre" – Study Baseline; "D" – day number preceding first cycle (D-) or following (D+) carboplatin ("C") administration; "3M" – 3 months after initiation of therapy. Values are expressed as means and 95% CIs.

All 25 patients were evaluated for the hematopoietic effects of chemoimmunotherapy. Chemistry analyses were part of ongoing safety evaluations and are not reported here in detail. The white blood cell (WBC) count increased as expected after each GM-CSF cycle and peaked 6 days after the first dose of carboplatin (Figure 2A shows means with 95% confidence intervals). The neutrophil and eosinophil curves followed that of the WBC. The MO count increased considerably after the first immunotherapy dose of GM-CSF and IFN-γ1b and on day 9 compared with day -8 (Figure 2B). Prechemotherapy platelet counts were statistically unchanged throughout the 3-month study period, although they did increase significantly on day 9 of the second (*P *= .002) and third (*P *≤ .04) treatment courses (Figure 2C). When we compared the values obtained on day -8 with those obtained on day 9, there were significant increases in total WBC, neutrophil, MO, and eosinophil counts at day 9 (*P *≤ .001 for all comparisons) (Fig. 2A–C).

### Adjustments to treatment regimen

Sixteen of the 25 (64%) patients required a dose escalation of GM-CSF from 400 to 600 μg. Twenty of the 25 (80%) patients met the target MO expansion of a doubling from day -8 and >1,000 cells/μL. For two patients, the dose was reduced from 600 to 400 μg because of toxicity, one before and one after the second course. In five of the 25 patients (20%), the presence of neutropenia or thrombocytopenia required that the day 7 dose of IFN-γ1b not be given (4 after 2^nd ^and 1 after 3^rd ^course).

### Immunologic cell alterations

**Figure 3:**
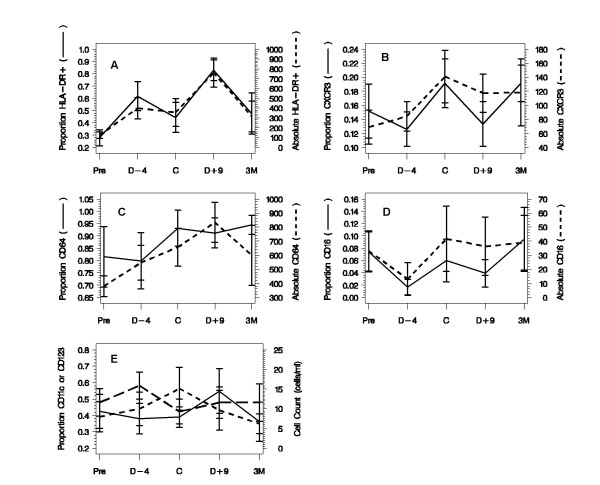
**Immunologic cell alterations as a function of time in 12 patients**. Proportions and absolute numbers of activated monocytes (HLA-DR+); low affinity FcR (CD16+); high affinity FcR (CD64+); chemokine receptor (CXCR3+); DCs (CD11c+ and CD132+). D4- = post GM-CSF dose; D9+ = post GM-CSF plus IFNγ . Values are expressed as means with 95% CI. Note: Solid lines represent proportions and short dashed lines represent total or absolute counts. In Figure E, a long dashed line represents the proportion of CD123 cells.

For 12 of the 25 patients (48%), MO and DC subsets from the first treatment course were examined as a function of time (Figure 3A–E). Compared with pretreatment, on day 9, there were significant increases in the absolute number of activated MOs, *i.e*,. HLA-DR+ cells (*P *= .04), cells with high-affinity Fc receptor (FcR), *i.e*,. CD64+ cells (*P *= .01), and cells with chemokine receptor, *i.e*,. CXCR3+ cells (*P *≤ .02). There were no significant differences in the absolute number of cells with low-affinity FcR (*i.e*,. CD16+ cells), the absolute number of DCs (*i.e*,. CD11c+ and CD123+ cells), or the proportion of DC subsets. In addition, there were no significant alterations in the numbers or proportions of natural killer (*i.e*,. CD3-CD56+) cells over the time period under study (data not shown). The proportions of CD83+ DCs and CD2+ cells at day -8 were 1.4% and 3.7%, respectively.

We also examined the CD32 MO population in a small sample of four patients. On day -8, the mean (± SD) number of CD32+ MO/MAs was 99.4 ± 0.4, and the mean fluorescent intensity (MFI) was 145 ± 51. On day 9, the mean number of CD32+ MO/MAs remained stable (99.9 ± 0.2) but the mean MFI increased to 250 ± 45.

CD32B expression was also measured in a small sample of two patients. The mean expression level was 36 ± 5% on day -8 and 37.4 ± 0.2% on day 9, and the corresponding mean MFI values were 16 ± 1% and 22 ± 1.4%.

### MO-mediated tumor cell cytotoxicity and ADCC

Using the SKOV3 cell line and a 20:1 effects-to-target ratio, we found overall lower *in vitro *MO-mediated tumor cell cytotoxicity and ADCC with purified MOs from EOC patients than with purified MOs from buffy coat samples of normal subjects (Table 2).

**Table 2:** MO-Mediated and Antibody-Dependent Cellular Cytotoxicity of Untreated MOs from EOC Patients and Normal Subjects

	EOC Patients		Normal Donor Samples*		
		
MO antibody treatment	No.	Mean ± SD	No.	Mean ± SD	*P *Value
None	8	0.8 ± 1.9	4	3.4 ± 3.2	.04
2B6 antibody	6	1.0 ± 2.3	4	8.5 ± 5.0	.02
trastuzumab	8	2.5 ± 3.1	5	11.5 ± 11.1	.06
trastuzumab + 2B6 antibody	6	4.1 ± 4.4	6	9.9 ± 5.9	.11

All experiments run with a concurrent control from 3 cryopreserved donor samples (see Methods).

Next, we compared the effect of GM-CSF treatment *in vitro *on MO-mediated tumor cell cytotoxicity and ADCC in samples from days -4, 1, and 9 of the first treatment course and 3 months after the first treatment course was begun with samples from pretreatment baseline (Table 3). There was no significant effect of trastuzumab treatment overall, although the number of patients tested was small and the standard deviations large. More patients are needed to assess whether GM-CSF treatment enhances the ability of MOs from blood to mediate cytotoxicity or ADCC against the SKOV3 cell line and at later time points as tumor burden decreases in responding patients.

**Table 3:** Mean Percentage ± SD Cytotoxicity during Treatment against the SKOV3 Ovarian Tumor Cell Line

	Course 1				
		
MO Treatment	Day -8	Day -4	Day 1	Day 9	3 Months*
None	0.8 ± 1.9 (8)^†^	2.4 ± 3.6 (8)	1.3 ± 1.4(6)	2.8 ± 3.4 (7)	1.9 ± 0.9(3)
2B6 antibody	1.0 ± 2.3 (6)	2.3 ± 2.7 (6)	4.1 ± 3.2(4)	4.6 ± 3.0 (5)	4.6 ± 2.3(3)
trastuzumab	2.5 ± 3.1 (8)	3.7 ± 3.1 (8)	5.8 ± 4.7(8)	4.7 ± 5.4 (7)	4.3 ± 3.0(3)
trastuzumab + 2B6 a/b	4.1 ± 4.4 (6)	5.2 ± 3.3 (6)	10.8 ± 8.5(6)	7.6 ± 4.3 (6)	8.7 ± 5.0(3)

*Three months after first chemoimmunotherapy course was begun. ^† ^(n) = number of patients tested at each time period. *P *> .05 for day -8 vs each of the other categories (day -4, day 1, day 9, or 3 months).

### Plasma cytokine levels

Plasma levels of proinflammatory cytokines and the chemokine CXCL8 are shown in Table 4. During the first treatment course, (up to day 9), there were no statistically significant alterations in CXCL8 levels. M-CSF, also a product of MO/MA activation and which can be distinguished from GM-CSF by ELISA, was significantly increased after MO maturation and activation on D9. In contrast, at 3 months, plasma levels of IL-1α, IL-6, and IL-8 were significantly decreased (*P *≤ .033 for all three comparisons). GM-CSF levels were increased at each post treatment time point, suggesting that daily doses of GM-CSF were associated with its detection in the circulation.

**Table 4:** Plasma levels of cytokines and growth factors before and during 3 months of treatment.

**Cytokine or Chemokine**	Day*	**No. of Patients**	**Mean Concentration (pg/mL)**	**Change from Day -8 (pg/mL)**	**P value**^†^
IL-1α	-8	13	43		
	-4	12	47	5.2	0.224
	1	13	48	4.8	0.172
	9	13	48	4.6	0.437
	3M	11	29	-16.3	0.033
IL-1β	-8	13	109		
	-4	12	115	8.0	0.295
	1	13	122	13.6	0.052
	9	13	125	16.4	0.069
	3M	11	106	- 6.4	0.501
IL-6	-8	13	62		
	-4	12	76	12.3	0.166
	1	13	72	10.2	0.164
	9	13	79	16.3	0.094
	3M	11	50	-18.4	0.014
IL-8	-8	13	12		
	-4	12	15	3.0	0.074
	1	13	14	1.9	0.222
	9	13	13	1.2	0.463
	3M	11	8	- 4.6	0.019
IL-10	-8	13	9		
	-4	12	14	4.1	0.130
	1	13	11	1.7	0.149
	9	13	12	3.4	0.157
	3M	11	9	- 1.2	0.418
TNF-α	-8	13	150		
	-4	12	177	26.8	0.265
	1	13	174	23.4	0.200
	9	13	152	1.6	0.937
	3M	11	121	-39.8	0.377
GM-CSF	-8	13	2.9		
	-4	11	7.1	4.1	0.007
	1	13	3.5	0.6	0.012
	9	13	11.6	8.6	0.005
	3M	11	5.7	2.6	0.041
M-CSF	-8	13	519		
	-4	12	505	-26.0	0.847
	1	13	610	90.8	0.153
	9	13	863	343.9	0.007
	3M	11	558	13.6	0.902

*Days -8, -4, 1, and 9 of the first chemoimmunotherapy course; 3M, 3 months after the first chemoimmunotherapy course was begun.

^† ^P values not adjusted for multiple comparisons.

### Preliminary toxicities

All 25 patients were evaluated for up to 3 months for toxicity. Treatment with the cytokines GM-CSF and IFN-γ1b was well tolerated overall. Consistent with prior reports, 18 patients (72%) initially experienced grade 3 fatigue. The fatigue did not recur in subsequent treatment courses in any patient. One patient withdrew after the second treatment course because of grade 3 fatigue and chest tightness attributed to GM-CSF treatment. One patient who developed grade 1 liver toxicity after the third treatment course had to be removed from study after the fifth treatment course. A liver biopsy examination suggested drug-induced injury, but a viral etiology could not be excluded since there was serologic evidence of prior cytomegalovirus exposure. Six of the 25 patients required carboplatin desensitization for an allergic reaction in the second or third treatment course. Two patients experienced a severe allergic reaction to carboplatin and were removed from the study after the third and fourth treatment course, respectively.

### Quality of life

In the first treatment course, there was no significant change in FACT-G and BRM scores during cytokine administration before the infusion of carboplatin (FACT-G: day -8, 89; day -4, 86; day -1, 85; BRM: day -8, 54; day -4, 53; day -1, 52). On day 9, there was a statistically and clinically significant decrease in the FACT-G (90 *v *81; *P *= .009) and BRM (54 *v *49; *P *< .02) scores compared with day -8. The day before the start of the second treatment course, the FACT-G scores returned to pretreatment baseline values, but the BRM scores remained significantly suppressed (54 *v *50; *P *= .03). A similar pattern was seen for the third treatment course. Symptoms specifically related to BRM never returned to pretreatment baseline levels.

### Clinical response of patients through three treatment courses

Ten of the 25 patients (40%) had a clinical response, as based on measurable disease, after completion of three chemoimmunotherapy courses. Eighteen of these 25 patients (72%) had at least a 50% decline or normalization of the CA-125 level from pretreatment baseline through the end of the third treatment course, confirmed by two post-treatment readings.

## Discussion

As stated earlier, the purpose of this paper is to describe the main hematologic and immunologic effects observed in the initial 25 patients of the ongoing phase II chemotherapy clinical trial. Since the trial is ongoing, reporting of response and toxicity information should be considered preliminary. To facilitate the statistical analysis, we selected only the first 3 courses of treatment.

We have shown that 3 months of a prechemotherapy priming schedule of daily subcutaneous GM-CSF for 7 days followed by a postchemotherapy schedule of subcutaneous GM-CSF for 7 days was reasonably well tolerated. Increased absolute numbers of total WBCs, neutrophils and MOs were observed. GM-CSF is the only approved drug in the United States that can also stimulate non-granulocyte myeloid lineages such as MO/MAs and DCs.

The platelet count after three chemoimmunotherapy courses was not significantly altered from the pretreatment baseline count, but we thought it remarkable that there were statistically significant increases in platelet levels on day 9 of the second and third treatment courses. To our knowledge, ours is the first report of a significantly higher platelet count after GM-CSF treatment than before GM-CSF treatment. This increase occurred at the same time as the counts of the myeloid lineages were increased and the levels of IL-6, which has platelet-stimulating properties, were decreased. An early study had reported higher platelet levels in a group of patients treated with postchemotherapy GM-CSF than in another patient group not treated with GM-CSF after chemotherapy[37] The frequency of carboplatin hypersensitivity was not higher than prior reports and probably reflects the number of prior carboplatin courses. [38] In contrast, treatment with macrophage colony-stimulating factor and IFN-γ has been shown to produce grade 4 thrombocytopenia, grade 3 hepatic toxicity, and exacerbation of chronic obstructive pulmonary disease[39]

In our study, the eosinophil count was increased on day 9 of all three treatment courses, but the count had returned to pretreatment baseline levels 3 months after the chemoimmunotherapy regimen had been started. An earlier study showed no association between the occurrence of hypersensitivity reactions to carboplatin and eosinophilia.[37]

One of the aims of our study was to determine whether priming and postchemotherapy treatment with cytokines GM-CSF and IFN-γ1b increases MO counts. We found that MO counts increased significantly on day 9 of each of the three treatment courses and that MO values almost doubled from day -8 to day 9 of each treatment course. Eighty percent of the patients met the criteria of a doubling of the MO count and >1,000 MOs/μL. From our results, daily subcutaneous administration of 400 μg of GM-CSF appears to be a reasonable starting dose and schedule in this previously treated patient population. In another study, although daily and twice daily GM-CSF dosing over 10 to 14 days was administered after carboplatin and cyclophosphamide treatment, the addition of a twice daily prechemotherapy priming schedule for 4 days resulted in unacceptable toxicity in a small cohort of patients.[40]

IFN-γ is the most potent clinically available activator of MO/MAs. In adaptive immunity, IFN-γ participates in the conversion of precytotoxic to cytotoxic effector T cells. IFN-γ also enhances natural cell activity and can inhibit tumor cell proliferation directly by a variety of mechanisms[41] An *in vitro *study has suggested that IFN-γ plus IL-4 can induce maturation of myeloid DCs to a predominantly CD11C^+ ^phenotype after GM-CSF administration[42] We did not observe statistically significant alterations in DC numbers or maturation. IFN-γ -activated MDM MOs may contribute to the destruction or inhibition of tumor cells by releasing tumor-inhibitory molecules or by direct surface cell-to-cell interactions, such as ADCC or phagocytosis, using the Fc-γ R pathway. Activated MO/MAs can produce oxygen radicals and nitric oxide. In an early study, subcutaneous administration of IFN-γ, even after a single dose of 100 μg, increased H_2_O_2 _production and Fc-γ R expression by MOs, whereas clinical toxicity was low[43] In other studies, intravenous IFN-γ at doses up to 100 μg also induced MO secretion of H_2_O_2_,[44] although higher doses of IFN-γ (500 μg or higher by continuous infusion) were associated with immunosuppressive effects[45] We considered the 100-μg subcutaneous dose appropriate for combination with GM-CSF in the current study. IFN-γ has also shown activity against ovarian cancer in preclinical models[46] and in patients receiving intravenous or intraperitoneal injections[47,48] A recently reported randomized trial of cisplatin and cyclophosphamide plus IFN-γ (administered subcutaneously at a schedule of 0.1 mg for six doses per chemotherapy course) resulted in increased time to tumor progression among ovarian cancer patients and increased survival compared with cisplatin and cyclophosphamide alone[49] Because of the antiproliferative activity of IFN-γ, dividing cells such as bone marrow progenitors might be susceptible to frequently repeated systemic treatments. Despite our limiting rIFN-γ1b to four doses per treatment course, the dosing had to be reduced to one per GM-CSF cycle in five susceptible patients.

GM-CSF plus rIFN-γ1b also produced alterations in the activation cell surface marker phenotype of the MO population. Compared with pretreatment baseline, the proportion and number of HLA-DR+ cells increased significantly after GM-CSF treatment alone (day -4) or with IFN-γ1b (day 9) (Fig. 3A). The numbers of CD64+ cells (*i.e*,. cells with high-affinity FcRs) also increased, but there were no significant changes in CD16+ cells (*i.e*,. cells with low-affinity FcRs). In contrast, the mean fluorescent intensity (MFI) of CD32+ cells, which made up 100% of MOs before treatment with GM-CSF, increased significantly. Two previous studies had suggested that GM-CSF can stimulate antitumor functions, such as ADCC, or MO-mediated cytotoxicity[17,50] We found that after the administration of GM-CSF and rIFN-γ1b, there was an increase in the total number of MOs and activated subsets. However, there was no statistically significant enhancement of the MO-mediated cytotoxicity or ADCC effect after treatment. These results might be due to the fact that the CD16 low-affinity FcR is expressed on only about 10% of MOs and was not significantly altered; however, MOs also express other low-affinity FcRs, including CD32 and its allotypes CD32A and CD32B. CD32A enhances ADCC, whereas binding of CD32B blocks ADCC[51] We have shown that CD32B, the inhibitory low-affinity FcR, is expressed by approximately 30% of MO/MAs. The reduced ADCC effect observed prior to the treatment compared to values from normals suggest that these patients might be exhibiting a defect in ADCC. We recently described a similar defective ADCC effect utilizing macrophage-derived monocytes (MDM) from blood and ascites of EOC patients [32], which is in agreement with the current findings. It is possible that the tumor suppressive effects might prevail, at least on certain MO-mediated antitumor activity, when tumor burden and consequently the cytokine suppressor environment is larger. This situation could be predicted to occur at earlier stages of chemotherapy induction or reinduction.

Previous work has demonstrated that systemic administration of GM-CSF increases numbers of intraperitoneal MAs, which could be important in ovarian cancer because EOC is primarily a tumor of the peritoneal cavity[12] In our study, the numbers of CXCR3+ MOs increased significantly on day 9 after administration of GM-CSF and rIFN-γ1b (Fig. 3). CXCR3, which is present on activated MO/MAs and on activated T cells,[52] binds several rIFN-γ1b- induceable chemokines, including CXCL9/Mig, CXCL10/IP-10, and CXCL11/I-TAC[53] These chemokines might facilitate recruitment of activated leukocyte subsets to the tumor site. IFN-γ- inducible chemokines may also induce the proliferation of vascular pericytes that may induce antitumor effects mediated through the common receptor CXCR3. The CXC chemokines are a family of molecules that can also regulate angiogenesis. The ELR+ molecules are generally considered pro-angiogenic, while the ELR- molecules are considered angiostatic[54] I-TAC has the highest affinity for CXCR3,[55] and rIFN-γ1b can upregulate the production of Mig, IP-10, and I-TAC[52] Tumor-associated MO/MAs have a range of effects that include both pro- and anti-inflammatory responses. Activation of MO/MAs by GM-CSF and IFN-γ might enhance antitumor activity, but might also secrete products that are proangiogenic or contribute to growth and metastasis[56,57] However, one might speculate that, as tumor burden decreases during chemotherapy, activated MO/MA might be polarized more towards antitumor effects as they are released from the influence of the tumor.

Measurements of cytokines can provide some indication of the patient's tumor environment and might also be useful for monitoring immunomodulatory alterations of the treatment. We showed here that there were no significant increases in the plasma levels of the proinflammatory cytokines IL-1α, IL-6, CXCL-8, or of TNF-α, even though these proteins are produced by activated MO/MA. This suggests that the combination of GM-CSF and IFN-γ1b may not directly enhance production of these proangiogenic cytokines *in vivo*. In contrast, the plasma levels of CXCL8 and IL1α were significantly decreased at 3 months as a number of patients (72% showed a decline in CA125 values) were beginning to show responses.

The preliminary response data for 25 patients showed that 6 experienced a complete response and 4 experienced a partial response. It was previously demonstrated that the length of an initial response to platinum-based chemotherapy in ovarian cancer is predictive of the duration of future responses[58] After completing accrual to the phase II portion of the current trial, we will determine whether this schedule of GM-CSF and IFN-γ1b plus carboplatin enhances progression-free survival over prior courses of treatment.

The quality of life component of the study demonstrated little change in general and BRM-specific symptoms due to the cytokine treatments. However, both aspects of quality of life were significantly affected by carboplatin treatment. General aspects of quality of life returned to pretreatment baseline levels between treatment courses, whereas the BRM-specific symptoms remained suppressed. This difference was most likely due to the residual effects of carboplatin. Understanding the quality of life effects of novel combinations of treatment is critical. Examination of whether the BRM-specific symptoms return to pretreatment baseline levels after treatment is ended will be important to determine.

In conclusion, this novel regimen of GM-CSF, IFN-γ1b, and carboplatin has an acceptable toxicity profile and significantly increases blood levels of activated MOs, platelets, WBCs, and neutrophils. Completion of the phase II study will provide data on response, time to tumor progression in responders compared with prior treatments, and toxicity and will provide an opportunity to determine whether ADCC activity recovers in responding patients.
